# Granzyme A Is Expressed in Mouse Lungs during *Mycobacterium tuberculosis* Infection but Does Not Contribute to Protection *In Vivo*

**DOI:** 10.1371/journal.pone.0153028

**Published:** 2016-04-07

**Authors:** Santiago Uranga, Dessislava Marinova, Carlos Martin, Julián Pardo, Nacho Aguilo

**Affiliations:** 1 Grupo de Genética de Micobacterias, Dpto. Microbiología, Medicina Preventiva y Salud Pública, Universidad de Zaragoza, C/ Domingo Miral s/n, 50009, Zaragoza, Spain; 2 CIBER Enfermedades Respiratorias, Instituto de Salud Carlos III, Madrid, Spain; 3 Servicio de Microbiología, Hospital Universitario Miguel Servet, ISS Aragón, Paseo Isabel la Católica 1–3, 50009, Zaragoza, Spain; 4 Immune Effector Cells Group (ICE), 3 Aragón Health Research Institute (IIS Aragón), Edificio CIBA, Biomedical Research Centre of Aragón (CIBA), Zaragoza, Spain; 5 Nanoscience Institute of Aragon (INA), University of Zaragoza, Zaragoza, Spain; 6 Fundación Aragón I+D (ARAID), Gobierno de Aragón, Zaragoza, Spain; Institut Pasteur, FRANCE

## Abstract

Granzyme A, a serine protease expressed in the granules of cytotoxic T and Natural Killer cells, is involved in the generation of pro-inflammatory cytokines by macrophages. Granzyme A has been described to induce in macrophages *in vitro* the activation of pro-inflammatory pathways that impair intracellular mycobacterial replication. In the present study, we explored the physiological relevance of Granzyme A in the control of pulmonary *Mycobacterium tuberculosis* infection *in vivo*. Our results show that, even though Granzyme A is expressed by cytotoxic cells from mouse lungs during pulmonary infection, its deficiency in knockout mice does not have an effect in the control of *M*. *tuberculosis* infection. In addition our findings indicate that absence of Granzyme A does not affect the protection conferred by the live-attenuated *M*. *tuberculosis* vaccine MTBVAC. Altogether, our findings are in apparent contradiction with previously published *in vitro* results and suggest that Granzyme A does not have a crucial role *in vivo* in the protective response to tuberculosis.

## Introduction

Tuberculosis is one of the leading infectious diseases affecting mainly developing and underdeveloped countries, and causing more than one million deaths per year. Thus, there is an urgent need to develop new preventive, treatment and diagnostic strategies which allow to efficiently reduce this widespread pandemia [1]. Comprehension of the mechanisms underlying the interaction between host immune system and *Mycobacterium tuberculosis* will aid in the rational design of such strategies to fight tuberculosis.

Granzymes (GZMs) are a family of serine-proteases whose expression has been classically associated with cytotoxic cells, mainly CD8+ and NK cells. Granzymes, especially granzyme B, play an important role in cell cytotoxicity [2]. Upon recognition of target cells, effector cells secrete granzymes via granule exocytosis, which reach the target cell citosol through membrane pores conformed by perforin oligomers. The observation that not all GZMs have cytotoxic capacity [3] and some of them like GZMA, GZMK and GZMM present pro-inflammatory potential [4, 5], has opened a debate in the last years about the non-cytotoxic roles of this family of proteases. GZMA is well recognized to be involved in inflammation, triggering the release of cytokines, such as IL1β, in macrophages. Indeed, absence of GZMA provides a considerable resistance to pro-inflammatory disorders like endotoxemia [5] and bacterial sepsis [6].

A previous work has analyzed the role of perforin and GZMB *in vivo* in the outcome of tuberculosis infection, finding that these molecules do not have a major contribution to bacterial clearance [7]. With regard to GZMA, several authors have described an increase of its protein levels in serum of patients with active tuberculosis [8, 9]. In addition, a recent study reported that inflammation induced by GZMA could impair intracellular mycobacterial replication in macrophages *in vitro* [10]. However, there is no data *in vivo* about the role of GZMA in the context of tuberculosis infection. In this work we analyzed the expression of GZMA in lungs from tuberculosis-infected mice and we used a mouse GZMA knockout strain to study the role of this molecule in protection against primary tuberculosis infection before and after vaccination with the live-attenuated *M*. *tuberculosis* vaccine candidate MTBVAC [11, 12].

## Materials and Methods

### Mycobacteria

Mycobacterial strains H37Rv [13] and MTBVAC [11] were grown at 37°C in Middlebrook 7H9 broth (BD Biosciences) supplemented with ADC (BD Biosciences) and 0.05% (v/v) Tween-80, or on solid Middlebrook 7H11 (BD Biosciences) supplemented with ADC. Bacterial suspensions for vaccination or infection were prepared in PBS from previously quantified mycobacterial glycerol stocks.

### Ethics Statement

Experimental work was conducted in agreement with European (Directive 2010/63/EU) and National (Real Decreto 53/2013) directives for protection of laboratory animals. All experimental procedures with animals described in this work were previously revised and approved by the “Comisión Ética Asesora de Experimentación Animal” (CEA) of the University of Zaragoza (Approved protocol number PI17/14).

### Mice

C57BL/6 wild type (Janvier Biolabs) or GZMA-knockout (provided by Markus Simon, [14]) mice were bred under specific pathogen free (SPF) conditions. GzmA-/- mice were originally generated as described by Ebnet *et al*. [14], and were backcrossed into the C57BL/6 background more than 10 times. Groups of eight- to twelve-week old male and female mice were intranasally challenged with low dose (100–150 CFU) of *M*. *tuberculosis* H37Rv in 40 μl of PBS. Four weeks later, mice were sacrificed and lungs were harvested for the subsequent analysis. To determine bacterial load in lungs, organs were homogenized and plated on solid agar medium. To characterize lung cell populations, lungs were incubated with DNAase I (Applichem) and Collagenase D (Roche) in order to obtain a cellular suspension. In vaccination experiments, mice were immunized subcutaneously with 10^6^ CFU of MTBVAC vaccine in 100 μl of PBS eight weeks prior to challenge. For survival experiments, mice were challenged intranasally with a high dose (1000 CFU) of H37Rv. Weight loss (assessed twice a week) and physical aspect (assessed daily) were monitored to evaluate disease progression and mice were humanely euthanized according to accepted pre-established endpoint criteria. These criteria were loss of 20% of body weight or physical changes associated with pain as appearance of piloerection or arched back. Animals were euthanized in all cases by CO_2_ inhalation. No animals died before meeting the established humane endpoint criteria.

### Flow cytometry

For cellular surface staining, cells were labelled with antiCD4-FITC, antiCD8-PE, antiNK1.1-PE, antiLy6G-Pacific Blue, antiCD11b-PE (BD Biosciences) or antiCD11c-FITC (Miltenyi Biotec) diluted in RPMI 1640 culture medium with 10% foetal calf serum (FCS). To determine GZMA-positive populations, cells were fixed and permeabilized with the Cytofix/Cytoperm Fixation/Permeabilization Kit (BD Biosciences) following manufacturer instructions. Cells were stained with antiGZMA antibody diluted 1:100 (provided by Markus Simon [15]), followed by incubation with a secondary antibody APC-conjugated anti-rabbit IgG (eBiosciences). To analyze IFNγ-producing cells, these were incubated overnight with Purified-Protein Derivative (PPD) 10μg/ml (Statens Serum Institute, SSI). Golgi inhibitor GolgiPlug (BD Biosciences) was added to cells during the last six hours of incubation. Cells were then fixed and permeabilized with the Cytofix/Cytoperm Fixation/Permeabilization Kit and stained with anti-IFNγ-APC (BD Biosciences).

### Statistical Analysis

GraphPrism software was used for statistical analysis. For experiments with two experimental groups, unpaired t-student test was used. When three or more groups were compared, One-Way ANOVA analysis with Bonferroni post-test was performed. Differences were considered significant at p<0.05.

## Results and Discussion

### GZMA is expressed in lungs from TB-infected mice.

**Fig 1:**
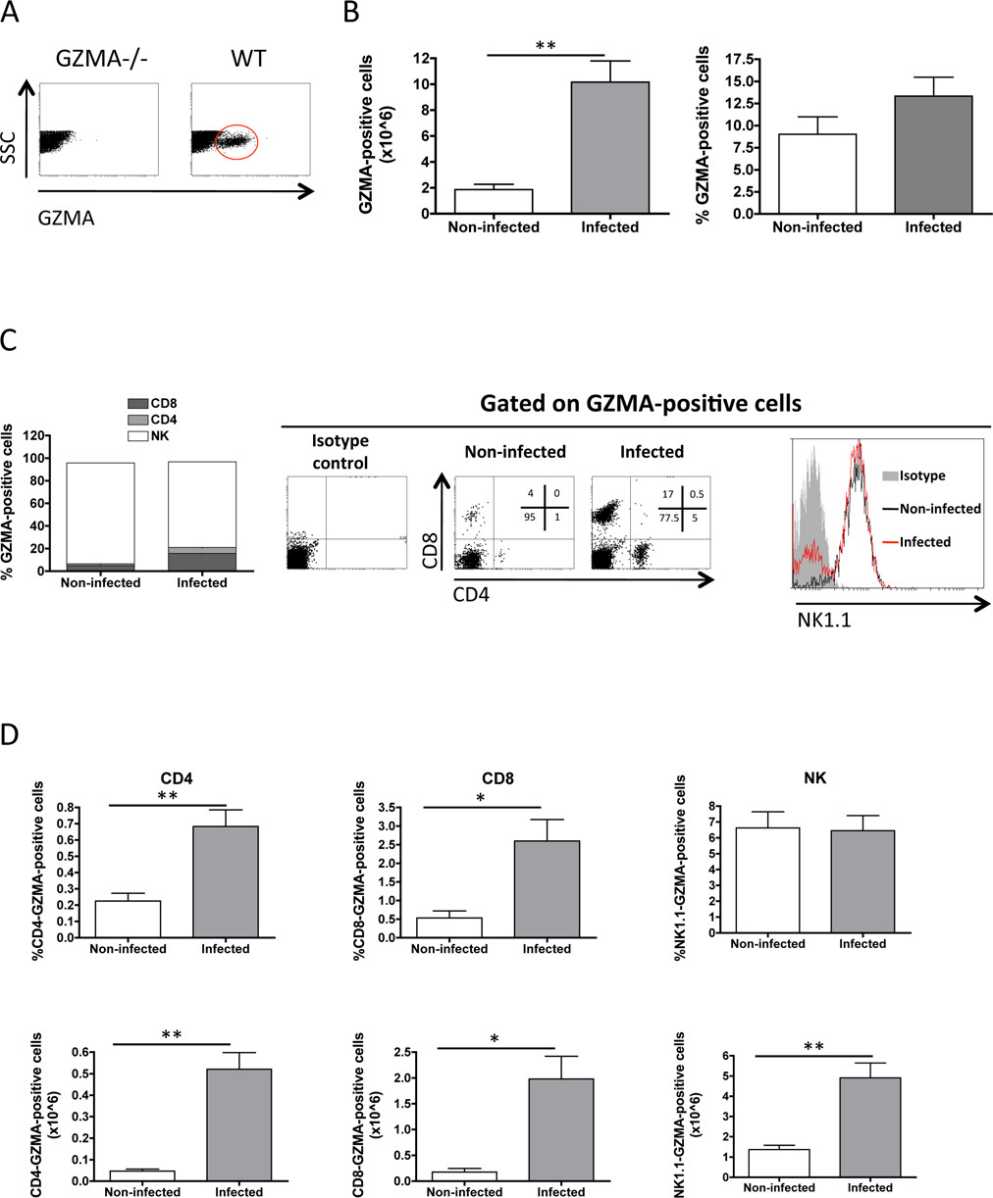
Granzyme A expression is increased in lungs after *M*. *tuberculosis* infection. **A**, lung cells from WT or GZMA-/- mice were intracellularly stained with anti-GZMA. A representative dot-plot showing GZMA staining is shown. GZMA-positive cells are contained in the red-circled region. **B**, **C**, **D**, Groups of five C57BL/6 wild-type mice were inoculated intranasally with a low-dose H37Rv challenge. Four weeks later, mice were sacrificed and GZMA expression analyzed in lung cellular populations. **B**, total GZMA-expressing cells. **C**, GZMA-expressing cell populations. Graph shows the relative proportions in percentage of the GZMA-positive cell populations. Right panels show representative CD4, CD8 and NK1.1 staining dot-plots and histogram gated from a GZMA-positive region. D, graphs show frequency (upper panels) and absolute number (lower panels) of CD4, CD8 and NK cells positive for GZMA staining, comparing non-infected and infected mice. A representative of two independent experiments is shown in the Figure. Data in the graphs are represented as mean ± SEM. Unpaired t-student analysis was performed to calculate statistical significance. * p<0.05; ** p<0.01; *** p<0.001.

To evaluate GZMA expression in lung cells, we first assessed the specificity of antibody used for GZMA detection by intracellular staining and flow cytometry. GZMA-positive cells were detected only in lung cells from wild-type (WT) but not from GZMA knockout (GZMA-/-) mice (**Fig 1A**). Our data revealed a significant 5-fold increase in the absolute number of GZMA-expressing cells in *M*. *tuberculosis* H37Rv-infected lungs compared to non-infected mice (**Fig 1B**). In terms of percentage, GZMA was expressed in approximately 9% of total cells in uninfected mice reaching more than 13% at four weeks post-infection. Then, we characterized by flow cytometry the GZMA-expressing cellular subsets in the GZMA-positive gated region (**Fig 1C**). Most of the GZMA-positive cells (> 95%) in non-infected mice were positive for the NK1.1 surface marker with a small proportion of CD8+ and CD4+ T cells, indicating that in the absence of infection GZMA expression in lungs is mainly restricted to NK cells. Conversely, four weeks post-infection, percentage of NK1.1-positive GZMA-expressing cells decreased to 80%, due to the substantial increase observed in the percentage of GZMA-positive CD8+ and CD4+ T cells, (15% and 5%, respectively). In concurrence with these observations, both frequency and absolute number of CD4+ and CD8+ GZMA-expressing cells dramatically increased in lungs upon infection (**Fig 1D**). Tuberculosis infection also led to an increase in the number of GZMA-expressing NK cells. In addition, we also analysed the presence of GZMA in neutrophils as expression of GZMA in human neutrophils has been reported [16]. However, we did not find GZMA-expression in mouse lung neutrophils (**S1 Fig**) confirming previous results in the mouse model [17].

Altogether, these data showed that an important number of GZMA-positive cells are infiltrated in *M*. *tuberculosis*-infected lungs, suggesting that this molecule could have a role in the outcome of tuberculosis infection.

### GZMA is not involved in protection against tuberculosis

**Fig 2:**
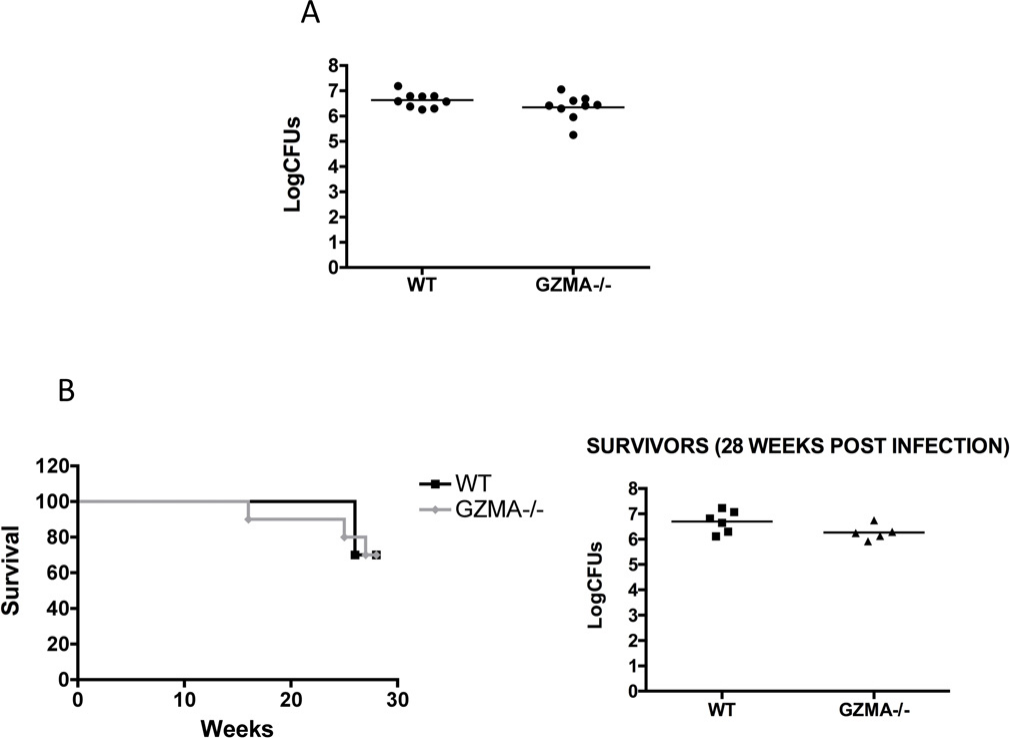
GZMA-/- mice are not more susceptible to *M*. *tuberculosis* infection. Groups of nine C57BL/6 wild-type or GZMA-/- mice were inoculated intranasally with a low-dose (**A**) or high-dose (**B**) H37Rv challenge. **A**, four weeks later bacterial burden in lungs was determined. A representative of two independent experiments is shown in the graph. **B**, animal survival (left panel) was determined according to pre-established endpoint criteria approved by an ethical committee. Data from one experiment are represented in a Kaplan-Meier survival curve. Lung bacterial burden in survivor animals was analyzed (right panel).

To elucidate the possible contribution of GZMA in the control of tuberculosis infection, WT and GZMA-/- mice were intranasally challenged with low-dose H37Rv. Four weeks after infection no difference in lung bacterial burden was found in WT and GZMA-/- mice (**Fig 2A**), suggesting no role of GZMA in controlling short-term infection. To assess whether GZMA could participate in controlling infection at long term, we evaluated survival after intranasal high-dose H37Rv challenge. As seen in **Fig 2B**, the survival rate of WT and GZMA-/- mice was similar at 30 weeks post infection, concluding that GZMA is not involved in long-term protection in our model. Consequently, no differences were found between both genetic backgrounds when analyzed lung bacterial burden in survivor animals (**Fig 2A, right panel**). Data shown in **S2 Fig** confirm no variation in lung cell populations (CD8, CD4, NK cells, neutrophils and dendritic cells) in GZMA-/- mice as compared to WT before and after tuberculosis infection. Our *in vivo* data contrast with a previous work showing that *in vitro* GZMA inhibits mycobacterial replication in macrophages [10]. This discordance could be explained by the difference in *in vitro* and *in vivo* conditions tested in both studies. In addition, our data do not necessarily exclude a role for GZMA in mycobacterial clearance, but that in its absence, other pathways could compensate its function.

**Fig 3:**
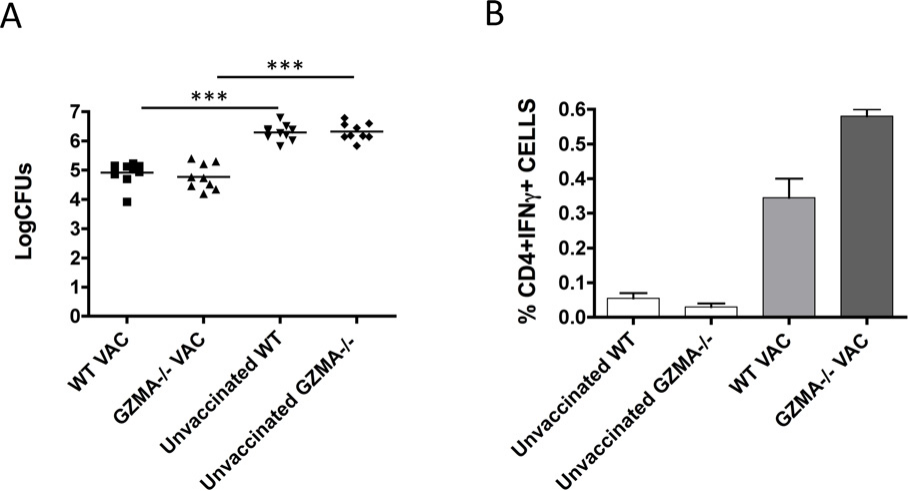
GZMA-/- mice are not less protected by MTBVAC vaccination. Groups of nine C57BL/6 WT or GZMA-/- mice were vaccinated subcutaneously with 10^6^ CFU of MTBVAC (WT VAC or GZMA-/- VAC), or non-vaccinated. **A**, at two months post-vaccination, mice were inoculated intranasally with a low-dose challenge of H37Rv, and four weeks later lung bacterial burden was determined. Data from one experiment are represented in the graph. One-way ANOVA test with Bonferroni post analysis was performed to calculate statistical significance. * p<0.05; ** p<0.01; *** p<0.001. **B**, cells were stimulated with PPD as described in materials and methods section, and CD4+IFNγ+ cells frequency in lungs was determined by flow cytometry. Data from one experiment are represented in the graph as mean± SEM.

Although we observed that GZMA is not involved in the control of tuberculosis infection, we sought to investigate whether it contributes to protection conferred by vaccination. To test this hypothesis, we used the live vaccine candidate MTBVAC [11, 12] for evaluation of protective efficacy against H37Rv challenge in WT and GZMA-/- mice. Our data revealed that vaccination reduced bacterial burden in both mouse strains to a similar extent, suggesting that GZMA is not relevant for vaccine-induced protective efficacy (**Fig 3A**). Interestingly, vaccination with MTBVAC induced similar PPD-specific IFNγ-producing CD4+ T-cell response regardless of GZMA presence; likely indicating that GZMA does not contribute to generation of the vaccine-induced protective response (**Fig 3B)**. Translating these data to clinical application of MTBVAC could suggest that vaccine-conferred protection would not be affected by potential loss-of-function *GZMA* gene polymorphisms.

Altogether, our data are in concordance with different works that show serum GZMA increase in patients with active tuberculosis [8, 9]. However, our results do not reveal any contribution of GZMA in the control of tuberculosis infection or in protective efficacy conferred by vaccination with MTBVAC. Thus, we suggest that the increase in GZMA expression observed in tuberculosis patients could be a consequence of immune system activation during the progression of disease rather than an effector mechanism to fight infection.

In some experimental models lack of GZMA has been shown to reduce susceptibility to inflammatory disorders like endotoxemia [4, 5] or bacterial sepsis [6] suggesting that GZMA-targeting inhibitors may be a potential attractive alternative to treat diverse inflammatory disorders. Our data suggest that potential use of GZMA-inhibitors would not increase risk of tuberculosis reactivation in latently-infected individuals, as observed in certain tuberculosis patients receiving anti-TNFα therapy [18] and as result have required tuberculosis prophylaxis treatment [19].

## Supporting Information

S1 Fig Granzyme A is not expressed by lung neutrophils. Groups of WT mice were infected intranasally with a low-dose challenge of H37Rv, or non-infected. Neutrophils were defined as CD11b+Ly6G+ cells, and analyzed in Granzyme A- negative- or positive- gated regions. A representative dot-plot of obtained from a non-infected and a tuberculosis-infected mouse is shown in the Figure.
(TIF)

S2 Fig Lung cellular populations in GZMA-/- mice after *M*. *tuberculosis* infection. Groups of WT or GZMA-/- mice were infected intranasally with a low-dose challenge of H37Rv. Four weeks later, lung cellular suspensions were prepared and CD4, CD8, NK cell, neutrophil (Ly6G^+^CD11b^-^) and Dendritic cell (CD11b^high^CD11c^high^) populations analyzed by flow cytometry. Data in the graphs compare infected and non-infected mice and are represented as mean± SEM.
(TIF)
