# Supplementary figures and images for: Granzyme A Is Expressed in Mouse Lungs during Mycobacterium tuberculosis Infection but Does Not Contribute to Protection In Vivo
Source: PLoS One. 2016 Apr 7;11(4):e0153028. doi: 10.1371/journal.pone.0153028 (PMC4824395; doi:10.1371/journal.pone.0153028)

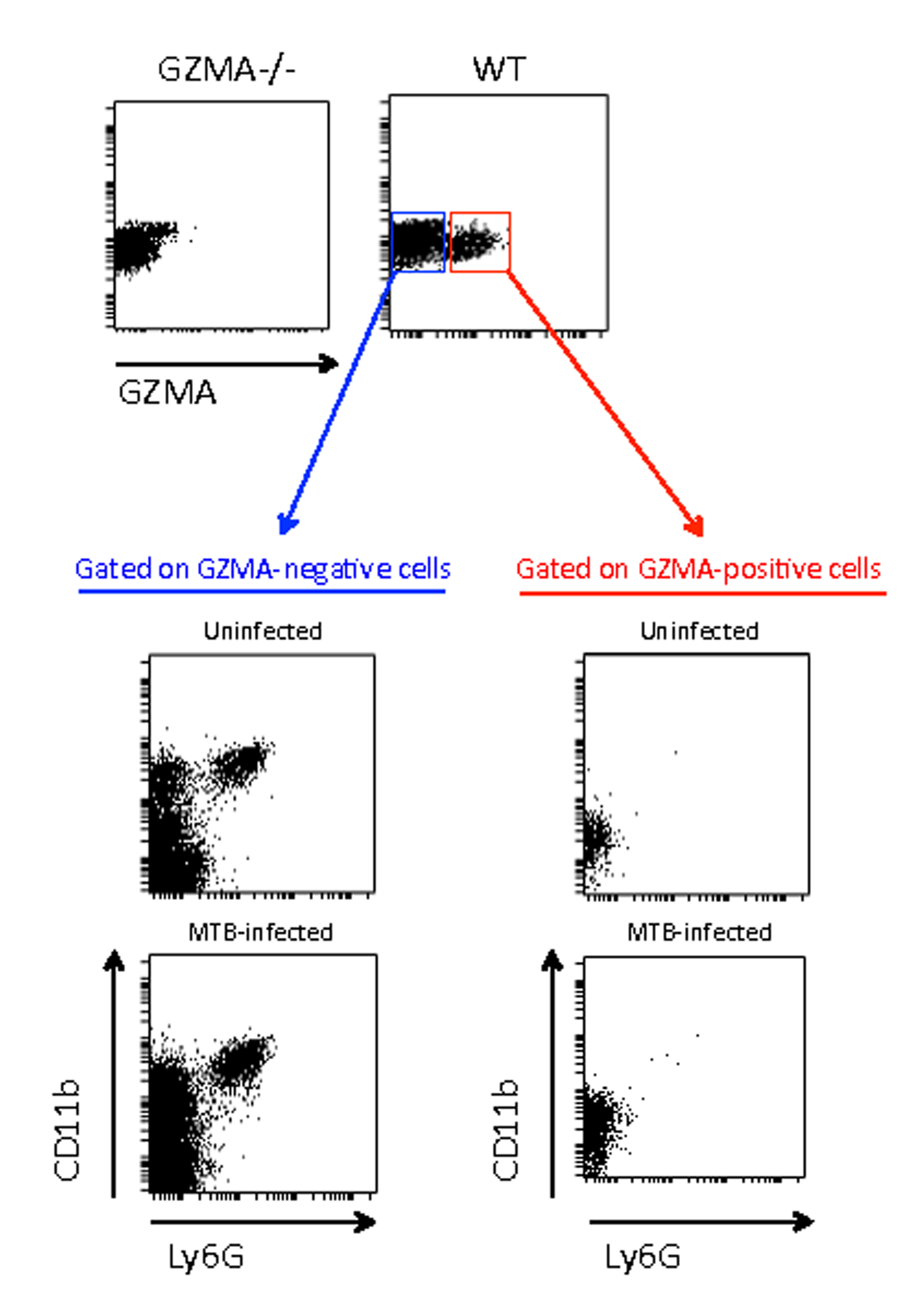

Supplement: S1 Fig — Groups of WT mice were infected intranasally with a low-dose challenge of H37Rv, or non-infected. Neutrophils were defined as CD11b+Ly6G+ cells, and analyzed in Granzyme A- negative- or positive- gated regions. A representative dot-plot of obtained from a non-infected and a tuberculosis-infected mouse is shown in the Figure. (TIF) [file pone.0153028.s001.tif]

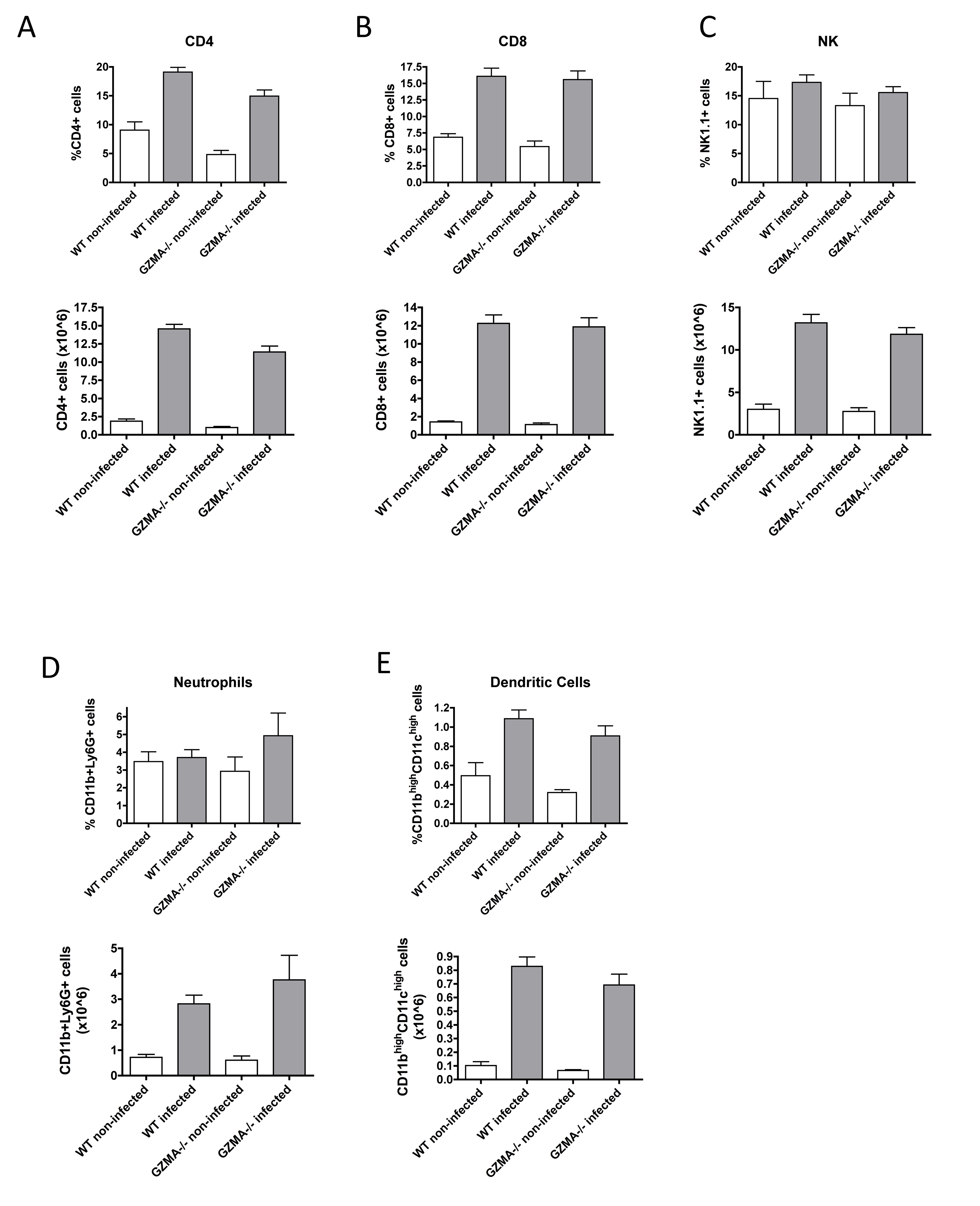

Supplement: S2 Fig — Groups of WT or GZMA-/- mice were infected intranasally with a low-dose challenge of H37Rv. Four weeks later, lung cellular suspensions were prepared and CD4, CD8, NK cell, neutrophil (Ly6G+CD11b-) and Dendritic cell (CD11bhighCD11chigh) populations analyzed by flow cytometry. Data in the graphs compare infected and non-infected mice and are represented as mean± SEM. (TIF) [file pone.0153028.s002.tif]
